# Do the Calls of a Bird, the Noisy Miner (Manorina melanocephala), Need Adjustment for Efficient Communication in Urban Anthropogenic Noise?

**DOI:** 10.3390/ani9030118

**Published:** 2019-03-26

**Authors:** Hélène Lowry, Alan Lill, Bob B. M. Wong

**Affiliations:** 1 School of Biological Sciences, Monash University, Clayton Campus, Victoria 3800, Australia; bob.wong@monash.edu; 2 Department of Ecology, Environment and Evolution, La Trobe University, Bundoora, Victoria 3083, Australia; A.Lill@latrobe.edu.au

**Keywords:** acoustic signals, anthropogenic noise, call adjustments, Noisy miner, urban adapter

## Abstract

**Simple Summary:**

A common feature of urban environments is constant, low frequency, anthropogenic noise. To communicate effectively acoustically in this environment requires avoidance of sound masking by this background noise. Research suggests that some animals can adjust their vocalizations so that they lie outside the main frequency range of urban anthropogenic noise, whilst others may produce sound signals that naturally avoid masking and therefore do not require adjustment. To date, research in this area has tended to focus on adjustment to complex songs, rather than simpler calls. The Noisy miner, *Manorina melanocephala*, is a very successful avian urban colonizer that uses calls in its acoustic communication. We compared the calls of this species in both urban and rural populations. Of all calls compared, only those that lay within the main frequency range of anthropogenic noise (1–2 kHz) showed shifts in minimum sound frequency in urban individuals, but these frequency shifts were notably small and insufficient to entirely preclude masking by urban noise. However, several Noisy miner calls had frequencies of more than 2 kHz that lay outside the main frequency range of urban anthropogenic noise (>2 kHz), suggesting intriguingly that this species may be inherently well-suited to communicating vocally in urban settings, which may be one of the keys to its success as an urban colonizer.

**Abstract:**

Urban environments are characteristically noisy and this can pose a challenge for animals that communicate acoustically. Although evidence suggests that some birds can make acoustic adjustments that preclude masking of their signals in high-disturbance environments such as cities, studies to date have tended to focus on acoustic signals important in mate attraction (e.g., songs). Far less attention has been given to the impact of urban noise on other kinds of calls. To redress this, we compared a range of different vocalizations (encompassing alarm calls, begging calls and parent response calls) among urban and rural individuals of a successful Australian ‘urban adapter’, the Noisy miner, *Manorina melanocephala*. We found that urban miners had significantly higher minimum sound frequencies for calls with low base-frequencies (<2 kHz); however, calls with base-frequencies ‘naturally’ above the main frequency range of urban noise (>2 kHz) had the same minimum frequency in urban and rural birds. Dominant frequency and call duration did not differ between urban and rural individuals. Although urban Noisy miners exhibited differences from rural individuals in the minimum frequency of calls, this shift was not large enough to avoid masking from low-frequency, anthropogenic noise. Nevertheless, our findings suggest that the calls of Noisy miners may be naturally well suited to being heard in noisy urban environments by having (a) dominant frequencies higher than low-level, anthropogenic noise and (b) several important call-types with frequencies above the main frequency range associated with urban noise.

## 1. Introduction

Anthropogenic noise is a common feature of urban environments and has the capacity to mask the vocal signals of animals that rely on acoustic communication. Indeed, recent observations suggest that anthropogenic noise may potentially exclude certain species from urban areas [1]. However, despite this, some animals, including many that rely on acoustic communication, are not only able to cope with a noisy environment, but appear highly adept at exploiting the conditions afforded by urbanization [2,3,4]. This ability can stem from them being ‘pre-adapted’ to the urban environment (e.g., [5]) or from them having the ability to adjust their behaviour to it (behavioural flexibility; [6]). The latter species are termed ‘urban adapters’ [3] and provide us with a unique opportunity to understand why some species prosper under human-altered conditions, whilst others do not [2,7].

Anthropogenic noise is most prevalent at lower sound frequencies (1–2 kHz), except for sudden, loud noises [8]. Such bursts of elevated environmental noise levels can be mitigated by a short-term behavioural response, such as temporarily halting vocalizing [9]. In contrast, ubiquitous, low-level noise poses a greater challenge in terms of vocal masking [10]. This is especially true for many birds that rely heavily on acoustic signals in social interactions, mate attraction, territory defense, antipredator behaviour and parent–offspring communication [11]. Evidence suggests that some birds in noisy, urban environments make vocal adjustments, such as alterations to frequency (kHz), timing, duration and/or amplitude of their songs. For example, urban European robins, *Erithacus rubecula*, reduce sound interference by singing at night in areas that were noisiest during the day [12], whilst the Common nightingale, *Luscinia megarhynchos*, increases the amplitude of its signals in the presence of fluctuating and chronically elevated noise levels [13]. Other research has found that urban Song sparrows, *Melospiza melodia*, [14] and Great tits, *Parus major*, [15] increase the minimum frequency of song notes and, in so doing, effectively reduce vocal masking in urban environments (see also [16,17,18] for other examples of frequency adjustments). There has been a substantial volume of research into facultative shifts in the vocalizations of urban birds, but most studies have focused on vocalizations important in mate attraction (e.g., songs). Surprisingly, far less attention has been given to calls that are produced in other important social contexts, such as alarm signaling and parent–offspring communication [1,18].

The Noisy miner, *Manorina melanocephala*, is a large (length 26 cm; mass 70–80 g), highly aggressive, communally-breeding honeyeater native to eastern and south-eastern Australia [19]. This species has recently successfully colonized and reached high population densities in urban environments [20]. In both urban and exurban environments, it aggressively excludes many other native bird species from the areas it occupies, a behaviour which has led some ornithologists to suggest radical, but controversial, solutions to conserve populations of the excluded species [21]. The Noisy miner is aptly named and easily identified by its distinct and persistent loud calls [22], making it an ideal species for a field-based acoustic study. The vocal repertoire of this species includes several alarm calls that appear to be functionally referential: The ‘aerial’ alarm call is primarily used in response to predators, whilst the ‘chur’ alarm call is used for less immediate, perching or terrestrial threats [23,24]. Additionally, the ‘cue’ vocalisation can often be heard during territorial disputes between conspecifics [25].

The aim of this study was to compare the pitch and sound frequency of calls produced by urban and rural Noisy miners. A variety of call types was investigated (i.e., alarm calls, begging calls, parent–offspring calls) to determine whether urban birds have diverged vocally from their rural counterparts. We were particularly interested in determining whether any such differences reduce masking by anthropogenic noise, or, alternatively, if Noisy miner vocalizations are inherently produced at higher frequencies than the low-frequency, anthropogenic noise typical of the urban environment (i.e., adjustment versus pre-adaptation).

## 2. Methods

Recording was conducted during a single breeding season from August to December. A colony was defined as a minimum of 3 Noisy miners separated by at least 1 km from any other group of conspecifics, but colonies usually comprised many more than three individuals. Colonies were located by playing back a Noisy miner chur alarm call (in uncompressed wav file format) broadcast through a hand-held speaker, as described by [26]. The alarm call was recorded using a hand-held directional Sennheiser RF-condenser microphone (MKH816TU, Wedemark, Germany) with a basket windshield (to reduce wind noise), connected to an M-audio micro-track 24/96 mobile digital recorder; the recording level was set at medium and the sampling rate at 44 kHz. Recordings of urban and rural Noisy miner alarm calls were obtained by entering areas known to be occupied by Noisy miners, which triggered alarm calling in response to the observer’s presence. Recordings of the most frequently heard alarm calls from multiple urban and rural sites were then cut-and-pasted into a coherent sequence using Raven Pro Interactive Sound Analysis Software (Cornell Lab of Ornithology, Ithaca, NY, USA) [27]. This was undertaken to ensure that focal birds from both habitat types were responding to a recording that was not biased toward urban or rural sites, in case there were differences in alarm calls between site types. Recordings were obtained from 140 colonies. These were equally divided between Metropolitan Melbourne, Victoria (37°50′ S, 145°00′ E) (70 urban sites), and the Central Victorian Goldfields (70 rural sites), encompassing the Maryborough (37°00′ S, 143°44′ E (165-km NW of Melbourne)) and Bendigo regions (36°40′ S, 144°15′ E (151-km NNW of Melbourne)). Urban sites were in parkland (multi-purpose sites comprising a combination of sports fields, playgrounds and public space, with varying proportions of native and exotic vegetation); rural sites incorporated National and State parks (open *Eucalyptus* woodland), as well as roadside habitat (*Eucalyptus* ‘corridors’).

### 2.1. Call Sampling

Recording of Noisy miner calls was conducted during the birds’ most vocally active period of the day (05:00–10:00 a.m.) [28]. Recordings were only made during dry, still conditions. Calls were recorded using the same recording equipment as that used to record alarm calls (see 2. Methods above). The recording level was set at ‘medium’ and the sampling rate at 44 kHz for both habitat types. On arriving at an occupied site, the observer located the closest out-of-nest juvenile, identified visually and by its distinctive begging call. The hand-held microphone was positioned so that there was clear air space between it and the vocalizing bird, thus limiting interference from background noise. All calls of juvenile(s) and associated adults were recorded for a period of 8 min. During a recording session, we identified the type of call and whether the bird was perching or flying. In instances where more than one juvenile was present during recording, each was given a number (spoken onto a digital recorder at the time of recording), so that individual begging calls could be identified later. A recording session was terminated if no vocalizations other than the begging call were obtained in 5 min or if weather conditions became unfavourable (i.e., wind velocity increased) or the focal juvenile(s) left the immediate area. In instances where a recording session was terminated before the 8-min limit, the site was revisited for a second session at least 10 days after the first visit.

### 2.2. Ambient Noise Sampling

To identify differences in ambient noise between urban and rural sites, ambient noise levels were recorded immediately after completion of call recordings, using the same equipment and settings as those used for recording Noisy miner calls. Ambient noise was recorded from the same location as the vocal recording. The observer first directed the hand-held microphone at the perching location of the calling bird and took a 1 min recording, then, following a circular track, recorded the bird at 90-degree intervals, until a total of four 1 min recordings had been obtained.

### 2.3. Analysis of Call Recordings

Calls were digitized at a 44 kHz sampling rate and 16-bit encoding using the M-audio micro-track 24/96 mobile digital recorder and were downloaded directly into Raven-pro 1.3 Beta Version Software (Cornell Lab of Ornithology, Ithaca, NY, USA), in which spectrograms (Hamming type) with a DFT (discrete Fourier transformation) length of 256 points were produced. Eight-minute recordings of calls were divided into 1 min sections. Random numbers were then generated to identify the 1 min section of the recording which would be analysed for a particular call type. We recorded and compared 5 different call types between urban and rural birds: The juvenile begging call, adult-to-juvenile post-feeding call and three different kinds of adult alarm calls (see [25] and introduction for alarm and territorial call classifications). Only one recording of each call type was analysed per study site. All call types analysed comprised a single, repeated note, except for the post-feed call that comprised two distinct call notes. The first clear note of the selected call type was identified visually for analysis based on the clarity of the call spectrogram; a pre-determined number of repeats (further renditions of the same note) of that call was also selected for analysis (begging call = 4 repeats, 2 alarm calls = 3 repeats each, 1 territorial call = 3 repeats, post-feed-call = single (note-a) single (note-b)). The selected call notes were then cut-and-pasted into a composite file. Visual analysis was conducted from spectrograms on all replicates in cut-and-pasted files, measured manually by drawing a selection around the chosen call note, where the top boundary is the highest frequency of the sound and the bottom boundary is the lowest frequency of the sound [27]. Ambient noise did not obstruct visibility of call spectrograms for any recordings, with calls being easily visible irrespective of overlap with ambient noise. Therefore, analysed calls were not biased toward lower ambient noise recordings. The following vocal components were measured: Minimum frequency (kHz), dominant frequency (kHz) (the frequency with the most energy) and delta time (s) (duration of call note). For each field site, measurements of the repeats of each vocal component for each call type were then pooled and mean values were calculated.

### 2.4. Analysis of Ambient Noise Recordings

Ambient noise recordings were digitized at the same settings as described for call recordings (see 2.3 Analysis of call recordings) and downloaded into Raven-pro 1.3 software (Cornell Lab of Ornithology, Ithaca, NY, USA). For each 1 min recording (4 × 1 min per site), four 2-s sections were randomly selected and cut-and-pasted into files. All noise events were included within this initial analysis, including vocalizations of heterospecific birds. A total of sixteen 2-s sections were obtained from each site, from which a site ambient noise mean value was calculated. For each section, the following features were analysed: Start time (s), end time (s) and frequency bandwidth (kHz). A second analysis of frequency bandwidth which excluded sudden, loud noises was also undertaken, as it was thought that constant noise would be the greater challenge for birds in terms of call-masking [10]. A single measure of the frequency bandwidth was obtained using the selection bar (see [27] for details on creating range selections in Raven-pro) to visually identify the frequency band, which remained constant throughout the entire 1 min recording. The four maximum frequency values for each site were pooled and a site ambient noise mean value was calculated. Recordings of both Noisy miner calls and ambient noise were analysed ‘blind’ as to the source of the recording (i.e., urban versus rural) to avoid any potential observer bias.

### 2.5. Statistical Analyses

All data were checked for normality and homogeneity of variances, but transformation was deemed unnecessary. Two sample t-tests were used to compare the calls of urban and rural Noisy miners, as well as the ambient noise levels between urban and rural sites, with the results being presented as mean ± SE and alpha set at = 0.05. Cohen’s *d* and the effect-size correlation were calculated where there were significant differences between urban and rural call frequencies and are presented in Hz and percentage terms. All analyses were performed using R version 2.2.0 (The R Foundation for Statistical Computing, Vienna, Austria).

### 2.6. Ethical Standards

The authors declare that all fieldwork described in this manuscript complies with the laws of Australia. Ethics approval for our study was granted by The Biological Sciences Animal Ethics Committee of Monash University (protocol no.: BSCI/2008/05).

## 3. Results

### 3.1. Call Features

**Figure 1:**
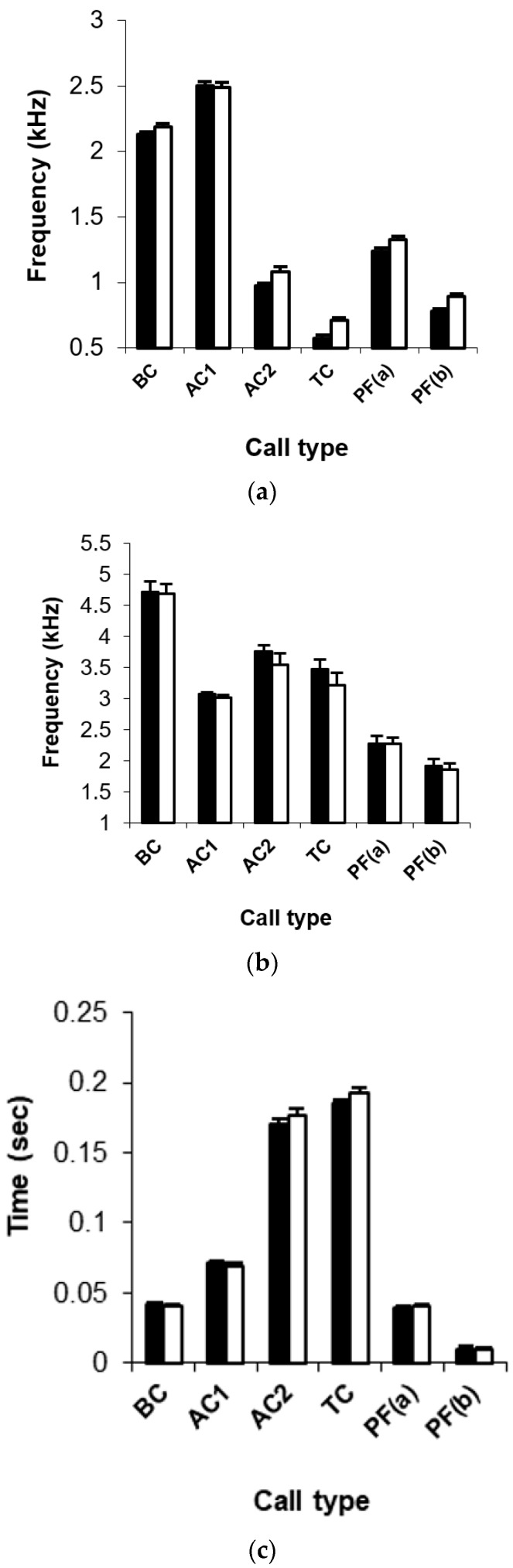
Comparison of three Noisy miner call features: (**a**) Minimum frequency, (**b**) dominant frequency and (**c**) call duration. Mean ± SE are given for five call types: (BC) Begging call, (AC1) aerial alarm call, (AC2) chur alarm call, (TC) cue territorial call, (PF) post-feed call (**a**,**b**) among rural (black bars) and urban (white bars) populations.

The minimum frequency of calls was not significantly different between urban and rural Noisy miners for higher base-frequency (>2 kHz) calls (begging call and aerial alarm call (A1); Figure 1a). However, calls with low base-frequencies (<2 kHz) had a significantly higher minimum frequency (*p* < 0.03) in urban than rural individuals (chur alarm call (A2) (mean difference: 109 Hz/14.7%) and cue territorial call (TC) (mean difference: 140 Hz/27.4%) and post-feed-call notes (1) (mean difference: 88 Hz/14.7%) and (2) (mean difference: 113 Hz/27.4%); Figure 1a). Dominant frequency did not differ significantly between urban and rural individuals for any call type (Figure 1b) Call duration did not differ significantly between urban and rural birds, except for the second note of the post-feed call, which was significantly longer in rural Noisy miners (Figure 1c).

### 3.2. Ambient Noise

**Figure 2:**
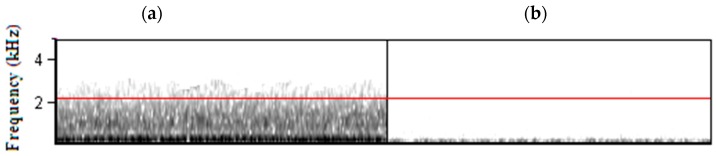
Spectrograms of ambient noise at (**a**) urban and (**b**) rural sites.

Overall, urban study sites had significantly higher maximum ambient noise frequencies (and consequently a broader frequency bandwidth) than rural sites (Figure 2). This was true irrespective of whether short-term, loud noises (defined as lasting <10 s) were included or excluded from the analyses (with short-term noises included: Means urban = 4.100 ± 1.193 kHz, rural = 2.519 ± 1.251 kHz, t = 7.549, *p* < 0.001; excluded: Means urban = 2.153 ± 0.551 kHz, rural = 0.956 ± 0.623 kHz, t = 11.972, *p* < 0.001, n_urban_ = n_rural_ = 70 in both analyses).

## 4. Discussion

### 4.1. Comparison of Call Features in Urban and Rural Habitats

#### 4.1.1. Minimum Frequency

**Figure 3:**
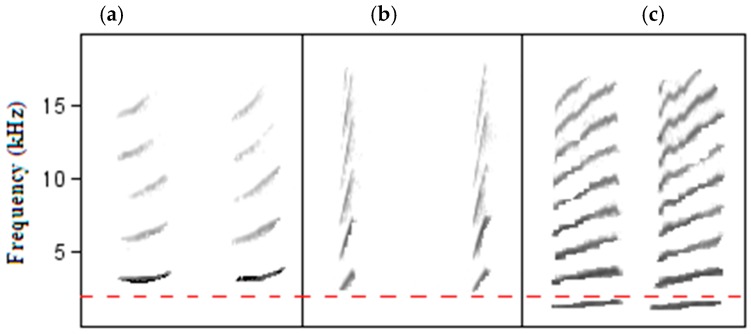
Spectrograms of three of the Noisy miner calls. (**a**) Adult aerial alarm call (AC1) and (**b**) juvenile begging call (BC) ‘naturally’ avoid low-level anthropogenic noise (1–2 kHz, marked by broken red line). (**c**) The lower frequency adult chur alarm call (AC2) does not avoid masking and showed a small shift in minimum frequency in urban birds.

We found significant differences in the minimum frequency of relatively low frequency call-types (<2 kHz) between urban and rural Noisy miners. In contrast, we found that Noisy miner calls with base-frequencies ‘naturally’ above the main frequency range of urban noise (>2 kHz) (see Figure 3a,b), as measured in this study (see Figure 2) and other studies (see [8]), had the same minimum frequency in urban and rural miners. In other words, there was no difference in calls that are expected to be robust against vocal masking. However, urban noise often extends beyond the described main frequency band of 2 kHz and thus one should not entirely discount the possibility that the higher frequency Noisy miner calls still overlapped spectrally to some extent with background urban noise. Facultative shifts in minimum frequency of songs have been identified in several songbirds [14,15,18,29,30] and, more recently, in calls in urban environments [18,31]. In these studies, birds were found to increase the lower frequency of signals above the main frequency band of anthropogenic noise and, in so doing, reduce vocal masking, although research suggests that these types of adjustments are not always sufficient to counter the effects of anthropogenic disturbance [32]. In contrast to other research in this area, a study investigating the alarm calls of Silvereyes, *Zosterops lateralis*, found that urban birds actually lowered the frequency of their alarm calls, effectively increasing the ‘active’ space of the signal, thus reducing masking from urban noise, which can vary in ‘energy’ within the 1–4 kHz frequency range [33]. Interestingly, the songs and contact calls of urban Silvereyes showed increased minimum frequencies [18], highlighting the need to consider differences in selective pressures on different signals [33]. Additionally, it may be important to consider the characteristics of the calls to which conspecifics pay attention (i.e., frequency versus duration of calls). Here, conservative call types might be favourable, irrespective of environmental noise, because the referential nature of the call is more important [23]. However, if simply hearing the call is key, and information is communicated to receivers through call structure, then it might be advantageous for a bird to be able to adjust the frequency of its calls in response to its environment. Interestingly, the significantly higher minimum frequency of the relatively low frequency call-types in the urban environment observed in the present study was not sufficiently higher to prevent masking of calls by background noise in urban habitats (see Figure 1a and 3c). A recent study of 12 species of urban birds encompassing both ‘songs’ and ‘calls’ found that species with intermediate minimum frequencies (1–1.5 kHz) raised the minimum frequency more than species with either higher or lower minimum frequencies [31]. Two of the three Noisy miner calls that we recorded that had a higher minimum frequency among urban than rural individuals had minimum frequencies within the intermediate range described by [31]. Interestingly, these authors found no difference between urban and rural Noisy miner ‘calls’, but, given that they combined all call-types together in a single analysis, it is possible that they simply were unable to detect the differences that we detected by analyzing different call types separately.

A possible explanation for the small shifts in minimum frequency observed in the current study (given the recent, large-scale, urban colonization by this species; [19], p. 626) is that vocal ‘adaptation’ to noisy urban habitats may still be in its early stages and may continue to evolve. Interestingly, small frequency shifts have also been observed in frogs inhabiting noisy environments. However, unlike songbirds, frogs do not learn their calls from conspecifics, and may therefore be expected to show a much slower population-level shift in call frequency (kHz) [34]. Alternatively, it has been suggested that an increase in song pitch might not be an adaptation that reduces sound masking by anthropogenic noise, but rather a physiological side-effect of birds signaling at higher amplitudes in urban environments [35]. Hence, the shifts in minimum frequency of calls observed in this study might simply be a side-effect of urban birds calling more loudly and recent research indicates that Noisy miners do indeed change the amplitude of their signals in urban noise [36]. However, the visual sound analysis method used for this study can be prone to measurement errors at finer scales and, therefore, the possibility of the small shift in minimum frequency being a false positive cannot be entirely discounted [37].

#### 4.1.2. Dominant Frequency

There was no discernable difference between urban and rural Noisy miners in dominant frequency of any of the calls measured. Shifts in dominant frequency have been identified in other birds [14,17,31] and frogs [34] inhabiting noisy environments. Notably, a meta-analysis by Roca et al. [38] found, from 36 studies compared, that birds typically demonstrated facultative shifts in dominant frequencies in the presence of anthropogenic noise, whereas anuran species mostly did not. These shifts are generally interpreted as a mechanism that reduces masking by low-frequency, anthropogenic noise, the dominant frequency being more important in this respect than other frequency parameters (e.g., minimum frequency). However, modifications to the dominant frequency may be costlier to the signaler [31]. Species with inherently high dominant frequencies might therefore be expected to have a ‘natural’ advantage in noisy urban environments. Research on 520 bird species’ vocalizations, encompassing ‘songs’ and ‘calls’, identified that species that are common in urban habitats generally vocalize at a higher dominant frequency than ex-urban, con-generic species [1]. A similar pattern has been shown for birds occupying roadside habitats; the songs of species that are more abundant near roads (indicating greater tolerance of noise) had significantly higher dominant frequencies than those of species that were less abundant near roads [39]. In the current study, the dominant frequency of all five Noisy miner calls was above the main frequency range of anthropogenic urban noise measured in this study (see results: Ambient noise), indicating that the calls are inherently audible in urban habitats. Higher dominant frequencies may, in part, reflect Noisy miners having calls with a broad bandwidth, with only the lowest harmonics of a call been masked. Hence, even for lower frequency calls, there is unlikely to be much selective pressure for this species to make vocal adjustments in urban environments. Interestingly, reductions in bandwidth, which can be a side effect of birds increasing the minimum frequency of vocalisations which reduces masking in urban noise, has been linked with poorer vocal performance in birds [40]. The high dominant frequency of Noisy miner calls probably reflects natural selection for higher frequency signals in open woodland birds. This is because attenuation of lower sound frequencies over distance is more pronounced in open habitats [41,42,43,44]. Thus, Australian open-woodland birds may be vocally ‘pre-adapted’ to inhabit urban environments, which may partly explain why open-woodland birds are such common residents of Australian cities [45,46,47,48].

#### 4.1.3. Call Duration

Increasing the duration of signals has been shown to improve the detectability of sounds in white noise [49] and several studies have found facultative shifts in call duration in birds living in noisy conditions in urban habitats [1,15]. In contrast, research on urban Song sparrows [14] and Dark-eyed juncos, *Junco hyemalis* [30], found that individuals did not change trill duration where both species showed adjustments to the frequency (kHz) of songs under noisy conditions. We found no differences in call duration between urban and rural Noisy miners, apart from the second note of the post-feeding call. Conceivably, Noisy miners lack the vocal plasticity to adjust the duration of call-notes. Alternatively, they may not need to adjust the duration of calls in urban environments because of ‘pre-adapted’ vocal traits, such as having dominant frequencies above urban anthropogenic noise averages (see dominant frequency). In this respect, inherent traits have previously been linked to a species’ ability to cope in urban conditions and have been highlighted as a possible explanation for a lack of variation between urban and rural con-specifics [50].

## 5. Conclusions

Urban Noisy miners exhibited differences in their calls from those of rural individuals that could reduce masking by low-level anthropogenic noise, but the shift in minimum frequency was not large enough to entirely avoid such masking. Call adjustments in this species may be at an early stage of ‘adaptive’ tuning to urban conditions, a process which may be slower for calls than for learned songs. Our findings also suggest that the calls of Noisy miners may be inherently well-suited to being heard in noisy urban environments by having (a) a dominant frequency above that of low-level anthropogenic noise and (b) several important call-types that are at higher frequencies than the main frequency range of urban noise. A detailed comparison of ‘calls’ of other conspecific urban ‘adapters’ and ‘avoiders’ would be required to establish this inference unequivocally. Temporal adjustments in calls (and associated behaviours) which are more ‘plastic’ and have been shown to improve signal transmission in ‘noise’ (e.g., increasing perching height during vocalizing; [51]) could also profitably be investigated as possible additional (or alternative) mechanisms employed by Noisy miners that mitigate urban noise effects.
